# Spike generation estimated from stationary spike trains in a variety of neurons *in vivo*

**DOI:** 10.3389/fncel.2014.00199

**Published:** 2014-07-25

**Authors:** Anton Spanne, Pontus Geborek, Fredrik Bengtsson, Henrik Jörntell

**Affiliations:** Neural Basis of Sensorimotor Control, Department of Experimental Medical Science, Lund UniversityLund, Sweden

**Keywords:** spike firing statistics, stochasticity, spinal interneurons, purkinje cells, golgi cells, molecular layer interneurons, synaptic integration, whole cell recordings *in vivo*

## Abstract

To any model of brain function, the variability of neuronal spike firing is a problem that needs to be taken into account. Whereas the synaptic integration can be described in terms of the original Hodgkin-Huxley (H-H) formulations of conductance-based electrical signaling, the transformation of the resulting membrane potential into patterns of spike output is subjected to stochasticity that may not be captured with standard single neuron H-H models. The dynamics of the spike output is dependent on the normal background synaptic noise present *in vivo*, but the neuronal spike firing variability *in vivo* is not well studied. In the present study, we made long-term whole cell patch clamp recordings of stationary spike firing states across a range of membrane potentials from a variety of subcortical neurons in the non-anesthetized, decerebrated state *in vivo*. Based on the data, we formulated a simple, phenomenological model of the properties of the spike generation in each neuron that accurately captured the stationary spike firing statistics across all membrane potentials. The model consists of a parametric relationship between the mean and standard deviation of the inter-spike intervals, where the parameter is linearly related to the injected current over the membrane. This enabled it to generate accurate approximations of spike firing also under inhomogeneous conditions with input that varies over time. The parameters describing the spike firing statistics for different neuron types overlapped extensively, suggesting that the spike generation had similar properties across neurons.

## Introduction

Synaptic integration, i.e., the process by which a neuron summates and transforms the information it receives from other neurons, has been extensively studied both *in vitro* and *in vivo* and can be described as variations of the original Hodgkin-Huxley formulations for conductance-based electrical signaling (Stemmler and Koch, 1999; Goldwyn and Shea-Brown, 2011; Drion et al., 2012). Hence, the process of synaptic integration, optionally extended with a cable-theory inspired multi-compartment model of the neuron (Rall, 1962), and the associated activation of possible active conductances can therefore be modeled to provide an accurate reflection of the membrane potential at the level of the soma and axon hillock for a single neuron. However, the process by which the time-varying membrane potential is translated into a train of spikes with the statistical properties that have been found *in vivo*, has proven more elusive. In principle, there are two different views, one that this process is deterministic and can be calculated with high precision using variations of the original integrate-and-fire principle, and another one where the concept of stochastic noise in the spike generation *per se* is providing a stochastic contribution to the times of spike initiation. Support for intrinsic noise can be found in recordings from neocortical pyramidal cells, where spikes can arise seemingly at random times from a flat membrane potential and where Hodgkin-Huxley models of different modifications fail to account for the spike initiation (Naundorf et al., 2006) (see however commentary published by Shu et al., 2006). Many other authors also agree that the spike generation mechanism *per se* is subject to noise (Schneidman et al., 1998; Averbeck et al., 2006; Faisal et al., 2008; Saarinen et al., 2008; Ozer et al., 2009; Richmond, 2009; Goldwyn and Shea-Brown, 2011). The stochastic component of the spike times can however also be the result of synaptic noise due from chaotic behavior inherent within the network interactions between otherwise deterministic neurons (Van Vreeswijk and Sompolinsky, 1996). Without a mechanistic explanation for the intrinsic neuronal noise, or access to the activity and structure of the local network of neurons, a description of the spike generation process can only be obtained empirically, by approximations. In the present paper, we aimed to provide such approximations based on long-term intracellular recordings from a variety of neuron types *in vivo*.

For models aiming to simulate the function of the neuronal circuitry, approximations of the spike generation need to be based on *in vivo* data. This is because the presence of synaptic noise *in vivo* changes the conditions for spike generation from the *in vitro* setting through the introduction of high-frequency fluctuations in the membrane potential (Gauck and Jaeger, 2000; Destexhe et al., 2001; Chance et al., 2002; Fellous et al., 2003; Suter and Jaeger, 2004; Destexhe and Contreras, 2006). However, different types of *in vivo* preparations will come with different types of complications for this type of data. For example, the use of anesthetics will introduce changes to the membrane responsiveness of the neurons and induce global patterns of cyclic variations in the spike firing (Destexhe and Sejnowski, 2003), which will make it difficult to find periods of statistically stationary spike generation. In the awake animal, these problems are eliminated but there could still be variations in global brain activity over time, as the result of internal brain processing reflecting e.g. emotional state or planned action, which could affect the spike firing statistics. To circumvent these possible caveats, we use the non-anesthetized, decerebrate preparation in which *in vivo* patterns of synaptic noise are maintained and variations due to changes in internal states are absent. With the *in vivo* whole cell recording technique, we obtain spike time series across a range of membrane potentials from a variety of subcortical neurons to approximate and model their spike generation. Surprisingly, even though these types of neurons are known to vary with respect to their intrinsic conductances, we found the statistics of their spike generation to be overlapping.

## Materials and methods

### Recordings of spike firing

The procedures of all experiments were approved in advance by the local Swedish Animal Research Ethics Committee (permits M32-09 and M05-12). Adult cats were prepared as previously described (Jorntell and Ekerot, 2002, 2006). Briefly, following an initial anesthesia with propofol (Diprivan ® Zeneca Ltd, Macclesfield Cheshire, UK), the animals were decerebrated at the intercollicular level and the anesthesia was discontinued. The animals were artificially ventilated and the end-expiratory CO_2_, blood pressure and rectal temperature were continuously monitored and maintained within physiological limits. Mounting in a stereotaxic frame, drainage of cerebrospinal fluid, pneumothorax and clamping the spinal processes of a few cervical and lumbar vertebral bodies served to increase the mechanical stability of the preparation. To verify that the animal was decerebrated, we made EEG recordings using a silver ball electrode placed on the surface of the superior parietal cortex. Our EEG recordings were characterized by a background of periodic 1–4 Hz oscillatory activity, periodically interrupted by large-amplitude 7–14 Hz spindle oscillations lasting for 0.5 s or more. These forms of EEG activities are normally associated with deep stages of sleep (Niedermayer and Lopes Da Silva, 1993). The pattern of EEG activity and the blood pressure remained stable, also on noxious stimulation, throughout experiments (see also Jorntell and Ekerot, 2006).

Spinal neurons (six separate animals) and cerebellar neurons (eight separate animals) were recorded in different experiments. For spinal recordings, a laminectomy was made to expose spinal segments C5-T1. For cerebellar recordings, the bony cerebellar tentorium was removed to expose the caudal part of the anterior lobe of the intermediate cerebellum, in order to access the forelimb region of the cerebellar C3 zone, where all cerebellar recordings were made. In both cases, a pool of cotton-in-agar was built around the recording regions. The dura was removed and the recording region was covered with paraffin oil to prevent drying of the tissue. In the case of spinal recordings, we also made small openings in the pia in order to facilitate penetration of the patch clamp pipettes. Also for spinal recordings only, the recording region was covered with agarose attached to the bone of the vertebrae in order to dampen tissue movements.

Patch clamp pipettes were pulled to 4–14 MOhm and contained a potassium-gluconate based solution, as previously described (Jorntell and Ekerot, 2006). The spinal neurons were recorded from the lower cervical segments (C6–C8) at depths of 1.8–2.8 mm, corresponding to laminae IV–VII. Since they were located above the level of the motor nuclei that contains the alpha-motorneurons, they were labeled spinal interneurons. The cerebellar cortical neurons were recorded from the superficial parts of the forelimb region of the C3 zone. Purkinje cells were identified by the presence of complex spikes. Molecular layer interneurons (MLints) were recorded from the superficial part of the cortex, visible from the surface, above the first Purkinje cell layer. Spiking neurons in the molecular layer were classified as MLints. Golgi cells were recorded from the granule layer located below the first Purkinje cell layer from the surface. They were classified as Golgi cells on basis of their regular spontaneous firing (Van Dijck et al., 2013).

For recordings of series of interspike intervals, we used a HEKA EPC 800 patch clamp amplifier set to current clamp. As the patch clamp pipettes approached a neuron we released positive pressure and applied gentle suction in order to establish a gigaohm seal before break-in. For all neurons, seal resistance varied between 0.8 and 5 GOhm. Suction and sometimes current command steps of one 1 nA were applied to obtain intracellular access. Access resistance was compensated for off-line and was usually between 20 and 80 MOhm. To change the firing frequency of a neuron we used either steady current injections lasting up to several minutes or current step commands lasting 500–4000 ms. For current step commands, when the neuron's stationary state (see below) bridged the time gap between two successive current step commands, the spike firing of the consecutive current step commands was treated as a single stationary state, omitting the interspike interval bridging the steps. The signal was converted to a digital signal using the analog-to-digital converter Power 1401 mkII from Cambridge Electronic Design (CED, Cambridge, UK). The neural responses were sampled at 100 KHz and recorded continuously with the software Spike 2 from CED. Spike2 was also used to identify the spikes and to obtain data of the spike time series. Power spectrograms of the spike firing frequencies were calculated using the multitaper method as implemented in the Chronux toolbox for Matlab (http://chronux.org/).

### Finding and evaluating the model

All raw spike trains used to construct the models were separated into shorter spike trains that were 50 spikes long, with a few exceptions where the original spike train was shorter than 50 spikes but longer than 40. Only trains that were found to have ISI distributions that were stationary and log-normally distributed were used further (see statistics section for details). All log-normal distributions were constructed as maximum likelihood estimates of the distributions of ISIs using the LOGNFIT method in MATLAB (MathWorks). The normalized input of each stationary region was then estimated by *x* = log(*i.s.d*). This value together with the firing frequency during the state was used to fit the frequency curve Equation (6b) by iterative least squares estimation using the MATLAB method NLINFIT. The normalized input was then estimated once again using the inverse of Equation (6b), *x* = log [exp (*E*^−1^/*c*_*x*_) − 1] − Δ_*x*_. The new estimate was used to evaluate the whether the means and standard deviation the models provided could describe the ISI distribution of the region using log-normal, gamma or inverse Gaussian probability density functions (PDFs). The fraction of regions that could not be rejected was seen as the model accuracy.

### Statistics

One reason for using the log-normal distribution over other commonly used distributions is the possibility to use tests for normality in order to select which spike-trains should be used to fit the model. We use the Shapiro-Wilk (SW) test (0.05 significance level), that have been found to have high statistical power for very short samples (Shapiro and Wilk, 1965). Long spike-trains can hence be divided into shorter trains that are used for the model.

Transient events that could affect the statistics of the spike-trains were avoided by only using regions that were found to be stationary by the Kwiatkowski, Phillips, Schmidt, and Shin (KPSS) (*p* < 0.05) test. Regions that were rejected by the KPSS-test were not used further.

Where ISIs have to be compared to a specific distribution (e.g., to evaluate the constructed model), we used the 2-sample Anderson-Darling (AD) test (Scholz and Stephens, 1987) instead of the commonly used Kolmogorov-Smirnov (KS) test, since the KS-test underestimates the test statistics whenever the mean and variance of the distribution is estimated from the empirical distribution and it is known to have poor statistical power (Shapiro et al., 1968; Stephens, 1974). The performance of AD is comparable to that of SW when the mean and variance is unknown (Stephens, 1974).

The local variability (LV) (Shinomoto et al., 2005), the third and the forth L-moment in the form of the L-skewness and L-kurtosis was used to compare the performance of the gamma, lognormal and inverse Gaussian distributions. The asymptotic approximations of the L-moments have been found to be reliable with sample sizes of 50 or more (Hosking, 1990). The confidence intervals of the constructed models were created by drawing a total of 50,000 spike trains consisting of 50 spikes each, where the input to the model was varied uniformly over the relevant input range (*X* ∈ [0, 9]). Linear regression using the Matlab method REGRESS was used to validate the constructed model. The coefficient of determination *R*^2^ is used as a measure to evaluate the constructed linear regression. From Equation (6a) the line should have the form log(*i.s.d*.) = *p*_1_ *x* + *p*_2_ where *p*_1_ = 1 and *p*_2_ = 0. For a valid set of model parameters, the confidence intervals of the parameters of the linear regression should envelope these values. In addition to this a, Durbin-Watson test (*p* < 0.01) was used to test whether the residuals of the linear regression were uncorrelated using the MATLAB method DWTEST. The SW-test was further used to test whether the residuals were normally distributed (*p* < 0.01).

## Results

The purpose of the present paper was to characterize and to mathematically describe the spike firing statistics of spontaneously active neurons in the non-anesthetized, decerebrate state. The first step was therefore to make whole cell recordings primarily from spinal interneurons but also Purkinje cells, molecular layer interneurons and Golgi cells of the cerebellar cortex in the decerebrated cat. The spinal interneurons were recorded from the lower cervical segments (C6-C8) at depths of 1.8–2.8 mm, corresponding to laminae IV–VII. The cerebellar cortical neurons were recorded from the superficial parts of the forelimb region of the C3 zone (Jorntell and Ekerot, 2002). In the decerebrate preparation, these neurons are all spontaneously active at rest.

### Data approximations

**Figure 1:**
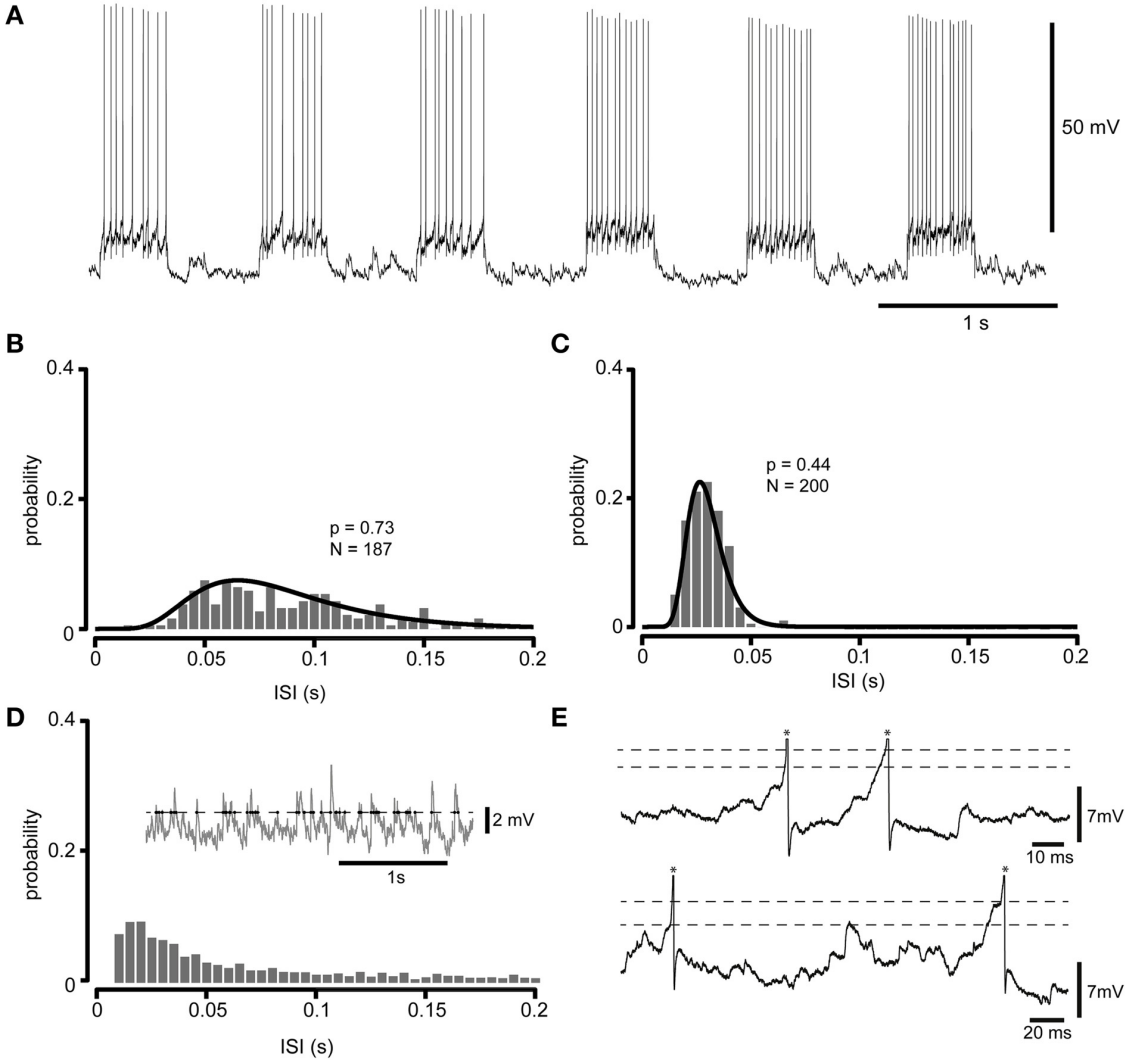
**Whole cell recording at multiple firing levels. (A)** Raw intracellular recording illustrating the spikes of a spinal interneuron recorded at rest and with two higher firing levels obtained through current injection (200 pA and 400 pA, respectively). **(B)** Frequency distribution histogram (5 ms bin width) of ISIs obtained by injections of 200 pA. The ISIs of all current injections at this level was pooled to construct the histogram. Also indicated is the log-normal distribution that provided the best fit for the ISI distribution at this firing level. **(C)** As in **(B)**, but for 400 pA injected current. In **(B,C)**, the *p*-value is given by the AD test, and indicates whether the null hypothesis that the ISIs originate from the log-normal distributions can be rejected or not. Since they are well above any confidence limit the found lognormal distributions can be said to well describe the ISI distribution. **(D)** For comparison, an analysis of the crossings of an arbitrary threshold level in the spontaneous synaptic activity. Same neuron as in **(A–C)**, but slightly hyperpolarized to prevent spiking. The inset show a raw intracellular sweep (2nd order Butterworth high-pass filter with a cutoff frequency of 1 Hz and a subsequent 2nd order Butterworth low-pass filter with a cutoff frequency of 1000 Hz; ISIs below 10 ms, which was not observed in this neuron (cf. **C**), were excluded for clarity) with a sample arbitrary threshold level (dashed line) and time points at which the membrane potential crossed from subthreshold to suprathreshold values (dots). The histogram illustrates the inter-event interval (IEI) distribution of such crossings. Note the much longer “tail” of membrane potential IEI distribution as compared to the ISI distributions in **(B,C)**. **(E)** Actual spike generation could occur at different membrane potential levels. Two sample raw traces recorded in the same neuron as in **(D)**, but without hyperpolarizing current injection, illustrate that the apparent spike (truncated, indicated by asterisks) thresholds (dashed lines, the threshold was defined as the point in time where the derivative of the membrane voltage exceeded a uniform threshold value) varied over time. Note the similarity with the findings of Naundorf et al. (2006) for neocortical neurons in that the spike initiation of our spinal interneurons also appeared to have a fast “kinked” onset with variable thresholds.

Our first aim was to analyze the distribution of the inter-spike intervals (ISIs) across the operative range of firing frequencies that these types of cells have previously been described to display under behavior (Edgley and Lidierth, 1987, 1988; Prut and Fetz, 1999; Pasalar et al., 2006; Takei and Seki, 2010, 2013; Badura et al., 2013). We used constant or episodic current injections to repeatedly modulate the neuron's firing frequency across these different levels, illustrated in Figure 1A for a spinal interneuron. During current injections we obtained spike firing levels during which the series of ISIs were stationary (KPSS-test, *p* < 0.05). The series of ISIs obtained at each firing level were binned in histograms (Figures 1B,C) to investigate the shape of the distribution of the intervals, which all had the skewed shape seen in the figures. Notably, the crossings of specific arbitrary threshold levels described by the subthreshold spontaneous synaptic activity had a different interval distribution than that of the spikes (Figure 1D), a discrepancy which could be explained by direct observations that the apparent spike threshold varied also at short time intervals (Figure 1E). In particular the long tail of distributions of the threshold crossing events in Figure 1D, which was absent in the ISIs, strongly suggests that the statistics of the spike generation was determined by other factors in addition to the synaptic inputs and the absolute value of the membrane potential. However, as the actual occurrence of a spike would inevitably affect the dynamics of the membrane potential (for example by absolute and relative refractoriness) in a way that we could not completely account for, we did not carry out the analysis described in Figure 1D in a systematic fashion.

The log-normal, the gamma and the inverse Gaussian distributions are typically used to describe skewed shapes of the type found for the ISIs. In this paper we focus on the log-normal distribution since it comes with the advantage that it is tied to an underlying normal distribution, which makes it possible to use tests for normality with high statistical power even with small sample sizes to validate the assumption that the ISI distribution can be described by a log-normal distribution (Shapiro et al., 1968). This is important since also temporary stationary states containing a limited number of spikes can then be used. Furthermore, the investigated neurons are typically participating in movement control of limbs where the phases of the movements have durations of at most a few hundred of milliseconds. Consequently, short spike trains can be useful also in this perspective as the aim of the model is to approximate the behavior of the neurons during natural circumstances. The log-normal distribution has previously been used to fit the spontaneous activity of neocortical neurons in the unanesthetized cat (Burns and Webb, 1976) and the spike output of detailed neuron models (Levine, 1991). As we will show below (**Figure 7**), the log-normal distribution provide at least as good approximations of the spike time distributions as the gamma and inverse Gaussian distributions.

The shape of the log-normal PDF in Equation (1) is determined by two variables, *μ* and *σ*, which corresponds to the mean and standard deviation of the distribution's natural logarithm, respectively.

(1) $$f\left(t;\sigma ,\mu \right)=\frac{1}{t\sigma \sqrt{2\pi }}\exp\left(\frac{-{\left(lnt-\mu \right)}^{2}}{2\sigma^{2}}\right)$$

**Figure 2:**
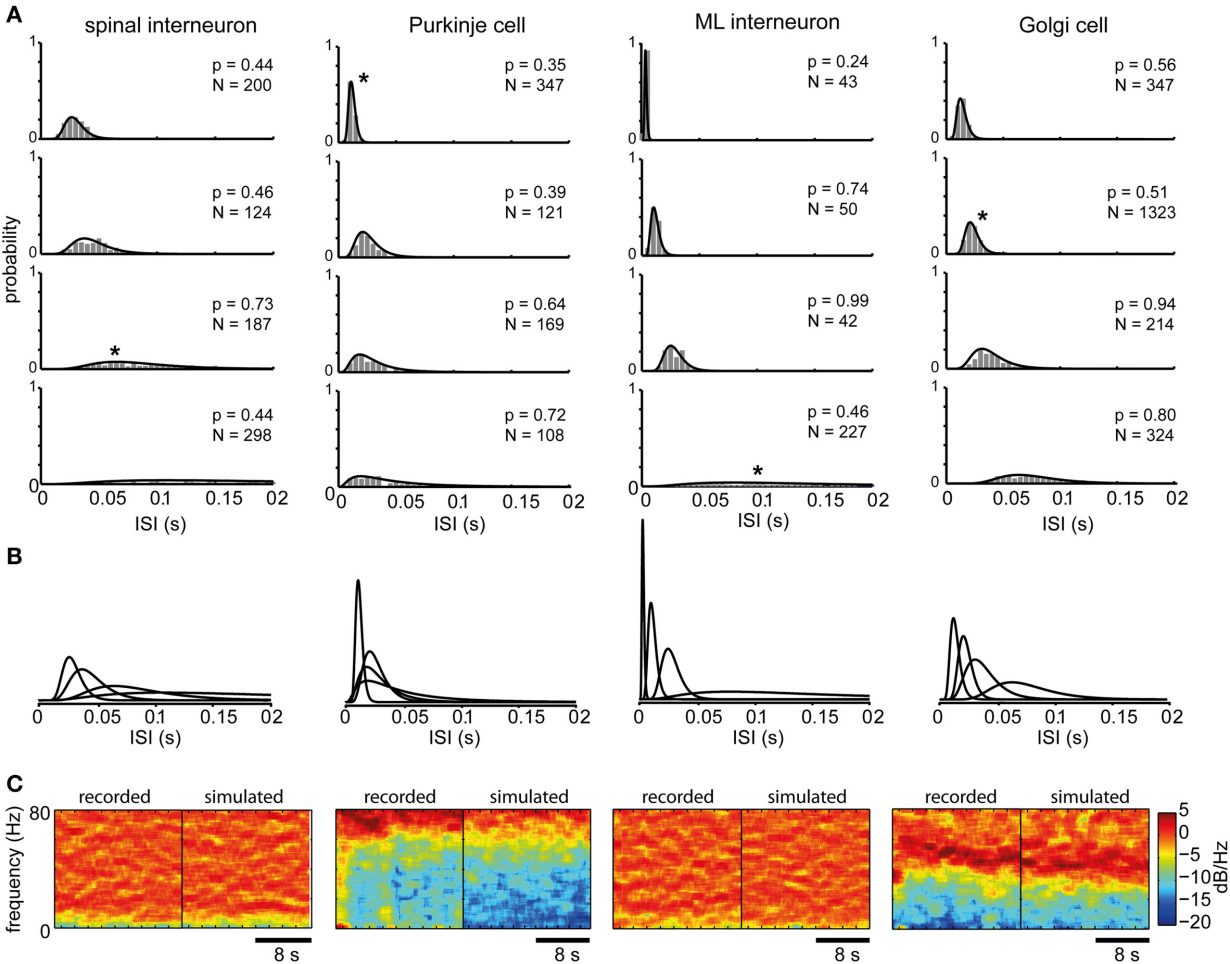
**ISI distributions at different firing levels for different types of neurons**. **(A)** The spike firing probability at four different firing levels (shown in ISI histograms with 5 ms bin width) and the maximum likelihood log-normal distribution for the cell type indicated at the top. Note that the x-axis is truncated at 200 ms, whereas many interspike intervals at the lowest firing levels could occur outside this range. N indicates the number of spikes in each histogram and the *p*-values above each histogram is given by the AD-test. A low *p*-value would mean that the null hypothesis that the ISIs originate from the log-normal distributions should be rejected, which is not the case in any of the shown distributions. **(B)** The fitted log-normal distributions for each neuron type are shown superimposed to facilitate comparison. **(C)** 20 s power spectrograms of the spike firing frequencies obtained from the same spike train data as the histograms marked with asterisks in **(A)** shown side-by-side with the power spectrograms of spike trains drawn from the point process defined by the corresponding log-normal distributions. These are shown by example to illustrate that we could not find any substantial cyclic variations in the overall spike firing frequency and that the frequency content of the recorded spike trains closely resembled that of the point process. Note that the large differences between neuron types are due to that they were recorded at different average firing frequencies.

The log-normal distribution provided a statistically good fit to the series of ISIs obtained at each firing level (Figures 1B,C). We also used the log-normal distribution to describe the spike firing statistics across different firing levels for single neurons (Figure 2). For spinal interneurons (spInt, *N* = 9, membrane resistance, *R*_in_, = 29 ± 11 MOhm) a total of 564 stationary states with log-normal ISI distributions were tested, corresponding to a mean of 63 ± 54 states per neuron. We also tested Purkinje cells (PCs, *N* = 3, *R*_in_ = 4.2 ± 1.2 MOhm), from which we obtained a total of 85 states (28 ± 21 states per neuron), molecular layer interneurons (MLint, *N* = 3, *R*_in_ = 132, 160, and 177 MOhm) with a total of 34 states (11 ± 8 states per neuron) and Golgi cells (Goc, *N* = 3, *R*_in_ = 30, 35, and 270 MOhm) with a total of 241 states (80 ± 46 states per neuron). Because the mathematical descriptions we apply below required a relatively high number of stationary states under stable conditions and for multiple levels of firing, we restricted our initial analysis to this limited set of neurons. In addition, to provide controls for the specificity of the model described later in the paper, we used the spike firing statistics of an additional 45 spinal interneurons.

The parameters of the log-normal distribution can be translated into the ordinary mean and standard deviation of the distribution through Equation (2):

(2) $$E=\exp\left(\mu +\frac{1}{2}\sigma^{2}\right)\;s.d.=E\sqrt{\exp\sigma^{2}-1}$$

where *E* is the mean ISI and *s.d.* is the standard deviation of the ISIs. By translating the parameters in this way, it is possible to relate them to the type of input frequency-current (f-I) curves that are routinely used to characterize neurons (Mckay and Turner, 2005; Molineux et al., 2005; Zhong et al., 2010). For this reason, we will use the inverse of the mean ISI to obtain the mean frequency *E*^−1^ below. We will also use the inverse of the standard deviation (*i.s.d*.) of the ISIs in order to keep the same unit for both measurements.

### Model

**Figure 3:**
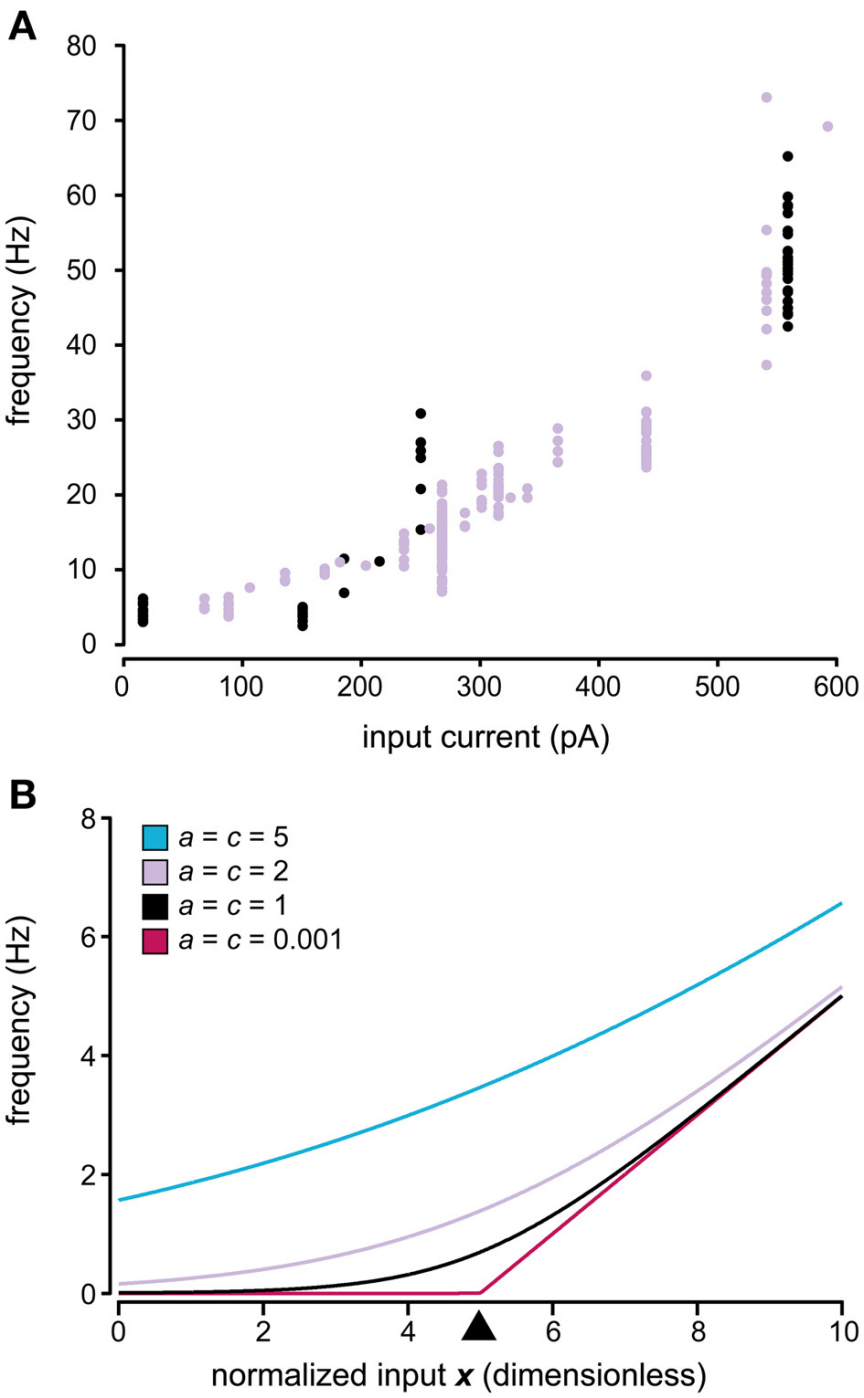
**Relationship between input and output in the model of spike generation**. **(A)** Relationship between injected current and the firing frequency for two spinal interneurons. Note the tendency of a smooth non-linearity rather than a sharp threshold as the frequency approaches 0 Hz. **(B)** Relationship between the input and the firing frequency using different parameters in the model of spike generation described in Equation (3). The values of parameters *a* and *c* are shown in the figure, where the value of c determines the width of the non-linear region. *a* = c in the figure to facilitate comparison between the curves as the slope of the linear asymptote, as the normalized input increases, equals *a/c*. The value of parameter *b* is set to 5, which means that the threshold is centered over 5, indicated by the black arrowhead. *b* has the same value in all curves shown. Note that both a sharp threshold is attained by having *c* close to 0, whereas a smoother transition is obtained with higher values of *c*.

Our next aim was to design a model describing the relationship between the input to the neuron and the statistics of its spike firing. The relationship between the input current and the spike firing frequency is frequently reported to be linear (Nowak et al., 2003; Mckay and Turner, 2005; Molineux et al., 2005; Meehan et al., 2010; Zhong et al., 2010). However, as the firing frequency of a neuron approaches zero the linearity inevitably disappears, either by a sharp threshold or by a smoother transition between zero and non-zero firing, also shown theoretically using leaky integrate-and-fire (LIF) neuron models (Fourcaud-Trocme et al., 2003; La Camera et al., 2008). Our results suggest a smoother transition in this non-linear region (Figure 3A), which is in agreement with other *in vivo* data (Priebe et al., 2004) and *in vitro* data stimulating the neurons with *in vivo* like inputs (La Camera et al., 2008), both obtained from neocortical neurons. It is possible that this property is more pronounced in an *in vivo* setting such as ours with an intense synaptic background activity as compared to *in vitro* since previous simulation work has suggested that the background level of stochastic synaptic input changes the dynamics of spike firing (Jaeger and Bower, 1999; Gauck and Jaeger, 2000, 2003; Destexhe et al., 2001; Salinas and Sejnowski, 2002; Fellous et al., 2003; Suter and Jaeger, 2004), especially close to the threshold (La Camera et al., 2008). A suitable description of the spike firing statistics should therefore be capable of featuring both a sharp and a smooth threshold in this region. At the high end it should be approximately linear, but also have a transition from the linear region to asymptotically reach 0 as the neuron is sufficiently hyperpolarized (Figure 3B). Here we introduce Equation (3), which satisfy these requirements. In this equation, the parameter *c* determines the width of the threshold region with *c* ~ 0 leading to a sharp threshold and higher values lead to a more smooth transition (Figure 3B).

(3) $$E^{-1}=a\ln\left[1+\exp\left(\frac{I-b}{c}\right)\right]$$

where *I* denotes the total input current over the membrane (i.e., the sum of synaptic and injected currents), *b* determines the position of the threshold and *a* the slope of the linear region. Due to the asymptotic linearity of Equation (3) at high input, it does not capture the high input saturation of the f-I curves obtained from LIF models (Rauch et al., 2003). However, none of the neurons we recorded displayed this behavior within the range of activity studied. It is possible that this behavior would have appeared if the neurons had routinely been driven to even higher levels of activity. However, when tested for a few of our neurons, at these levels we observed a spike break-down (severe reduction in spike amplitude, broadening of the spike) before the appearance of high input saturation.

**Figure 4:**
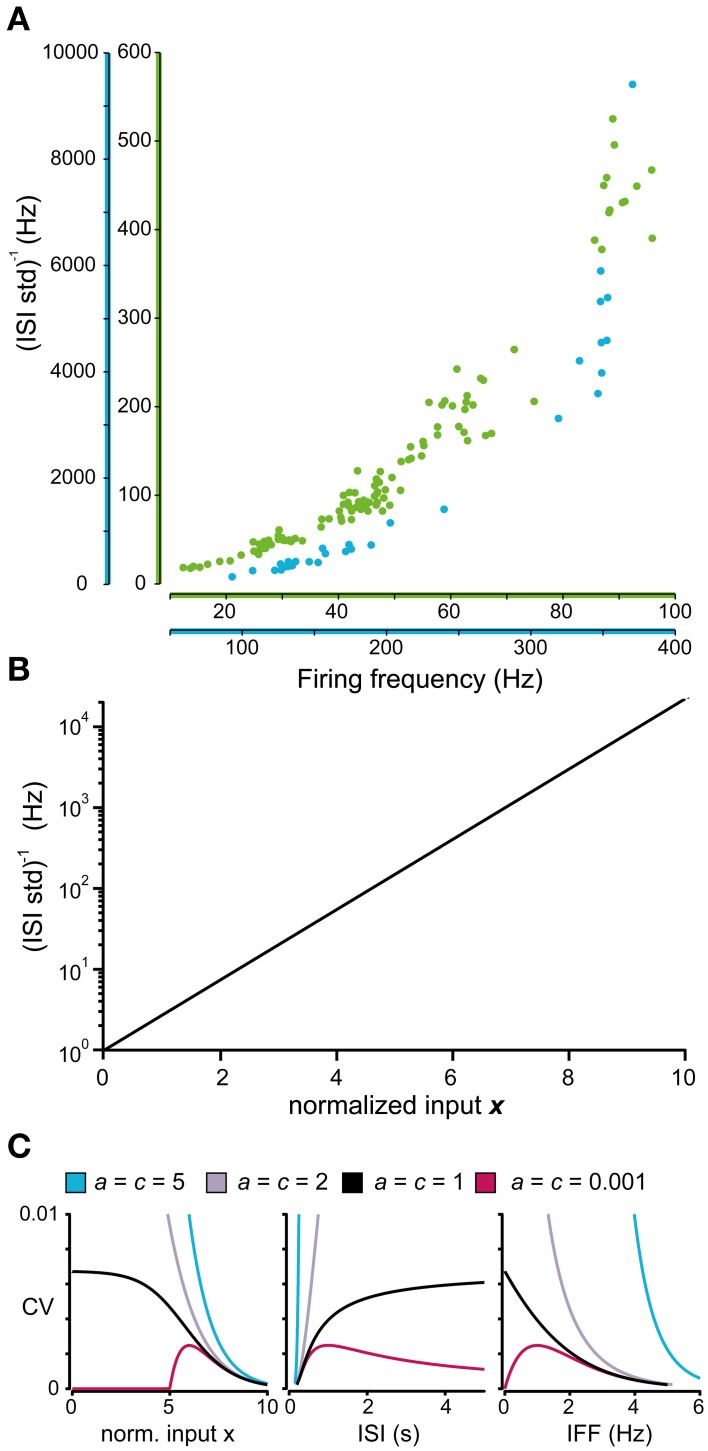
**Relationship between firing level and spike firing variability**. **(A)** Relationship between the firing frequency and the inverse of the standard deviation of the ISIs for two sample spinal interneurons. Note that the axes of the firing frequency for the two neurons differ substantially. **(B)** Model prediction of the relationship between the normalized input and the logarithmic value of the inverse of the standard deviation of the ISI distribution. **(C)** Predicted coefficient of variation (CV) of the complete model in Equation (6) where the parameters *a* and *c* are varied as in Figure 3. (**C**; left) Relationship between the normalized input and the CV. Note that the CV only reaches a stable value above 0 as the input approaches −∞ (corresponding to a substantial hyperpolarization) if *c* = 1. Otherwise it either diverges as *c* > 1 or approach 0 as *c* < 1. (**C**; middle, right) For comparison with previous results, the CV in relation to the average length of the ISIs (middle) (Softky and Koch, 1993) and the CV in relation to the instantaneous firing frequency (right) (Rauch et al., 2003) is also included in the figure.

An interesting feature became evident when we plotted the *i.s.d*. against the firing frequency. Rather than being linearly related, as would be expected from naïve rescaling of the ISI distribution, this relationship seemed to be best described as an exponential curve (Figure 4A). It is also clear from the two sample neurons in Figure 4A that the relationship was not the same for different neurons. A general exponential relationship between firing frequency and *i.s.d*. that could approximate both cases in Figure 4A is described by Equation (4):

(4) i.s.d.=exp(kI+m)

If the exponential curve appropriately approximates the relationship between the *i.s.d*. and input, it allows the model to be divided into two consecutive parts. The first part translates the input (e.g., input current) into a normalized dimensionless input *x* such that *i.s.d*. = exp *x*. Since all models must obey this relationship, it can be used as a method to validate the constructed models against the experimental data. By using a logarithmic scale, the exponential curve is transformed into the linear relation shown in Figure 4B, which simplifies visual inspection, but also enables the use of linear regression to validate the constructed model by testing whether the actual data are distributed along this line.

If the normalized input *x* is used instead of *I* in Equation (3), after modifying *b* and *c* accordingly, the coefficient of variation (CV) of the ISI over a range of input can be computed using Equation (5). Figure 4C illustrates how the CV varies using the different values of the parameters *a* and *c* that were also used in Figure 3B.

(5) $$CV=\frac{s.d.}{E}=a\ln\left[1+\exp\left(\frac{I-b}{c}\right)\right]\exp(-x)$$

During periods of high excitation, the *CV* will approach 0, as the ISIs will be defined by the properties of the neurons refractory period (Softky and Koch, 1993). The behavior during periods of low excitation is not as well defined. The parameter *c* of Equation (5) determines whether the *CV* approach 0 (*c* < 1), diverge (*c* > 1), or converge toward a fixed value (*c* = 1). The latter has support from studies of biophysical integrate-and-fire models (Softky and Koch, 1993; Rauch et al., 2003) while the other two options lack support or acceptable interpretations. From this, the parameter *c* should be set to 1 for the description of the firing statistics to have any meaning as the firing intensity approach 0. The actual asymptotic value of the *CV* can be calculated by the formula in Table 1.

**Table 1:** **Description of the parameters of Equation (6) and some related quantities**.

	**Description**
**Parameter**	
*c*_*I*_	Scaling between input current *I* and normalized input *x*
Δ_*I*_	Offset between input current *I* and normalized input *x*
*c*_*x*_	Scaling between the mean and standard deviation
Δ_*x*_	Offset between mean and standard deviation, center of threshold for *x*
**Quantities**	
*c*_*I*_(Δ_*I*_+Δ_*x*_)	Center of threshold for *I*
*c_I_c_x_*	*E*^−1^ ∝ *c_I_c_x_I* when *I* » *c_I_*(Δ_*I*_+Δ_*x*_)
*c_x_*e^−Δ_*x*_^	Coefficient of variation as *I* → −∞

The complete model can be summarized by the three equations in Equation (6). The first two Equations (6a,b) form a parametric equation between frequency and *i.s.d*. with the normalized input *x* as the parameter. In this way the model separated into one part that translates input to normalized input that is determined by the parameters Δ _*I*_ and *c*_*I*_, and one part that describes the relationship between frequency and *i.s.d*. that is determined by the parameters Δ _*x*_ and *c*_*x*_. The parameters and some related quantities are described in Table 1.

(6) $$\begin{array}{l} (a)\;i.s.d.=\exp x\;(b)\;E^{-1}=c_{x}\ln\left[1+\exp\left(x-\Delta_{x}\right)\right]\\ \;(c)\;x=c_{I}I-\Delta_{I} \end{array}$$

In summary, to find the parameters of the model that best describes the spike generation of a neuron from experimental data, one would need to find the mean and *s.d*. of the ISIs from several stationary states. These data can then be used to find the optimal parameters for that neuron in Equation (6).

### Fit between model and recorded data

**Figure 5:**
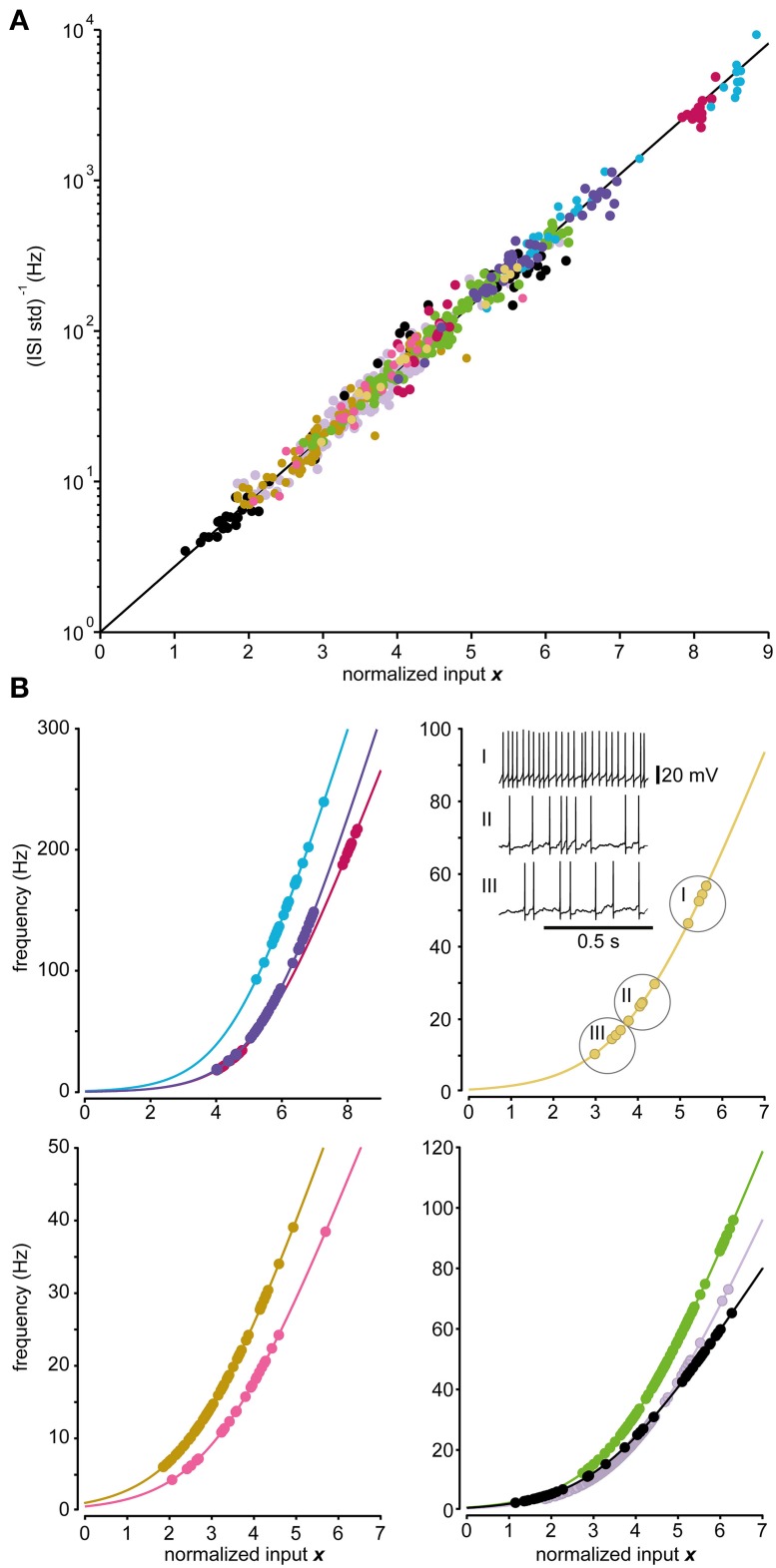
**Predictions of firing frequencies for spinal interneurons**. **(A)** Relationship between the normalized input and the logarithmic value of the inverse of the standard deviation of the ISI distribution for the model (line) and the recorded data of the 9 spinal interneurons tested. Each spinal interneuron is represented by a set of dots indicated by the same color. The recorded states are expected to lie upon the line according to Equation (6a). **(B)** Relationship between the normalized input and the firing frequency in the model (line) and the recorded data for the 9 spinal interneurons (dots) divided into 4 separate panels. The neurons are shown in different panels as they occupy different ranges of firing frequencies. Note differences in curvature and steepness of the curves between individual neurons. The inset displays three sample firing levels for one recorded neuron, as indicated by the encircled values in the curve.

Figure 5A demonstrates the fit of the spinal interneurons recorded to the relationship between input and the *i.s.d*. predicted by the model. None of the neurons failed the DW-test (*p* > 0.01) of the residuals and the linear regressions provided good fits (*R*^2^ = 0.96 ± 0.02; *N* = 9). In Figure 5B the relationship between input and spike frequency for each recorded spinal interneuron together with the fitted model of the spike generation for each respective neuron are shown. We further tested the accuracy with which the model could predict the distribution of ISIs for each stationary state. Using AD-test, we found that the model could predict the empirical ISI distribution using the lognormal distribution 97 ± 5% (*p* > 0.01, *N* = 564) of the states.

**Figure 6:**
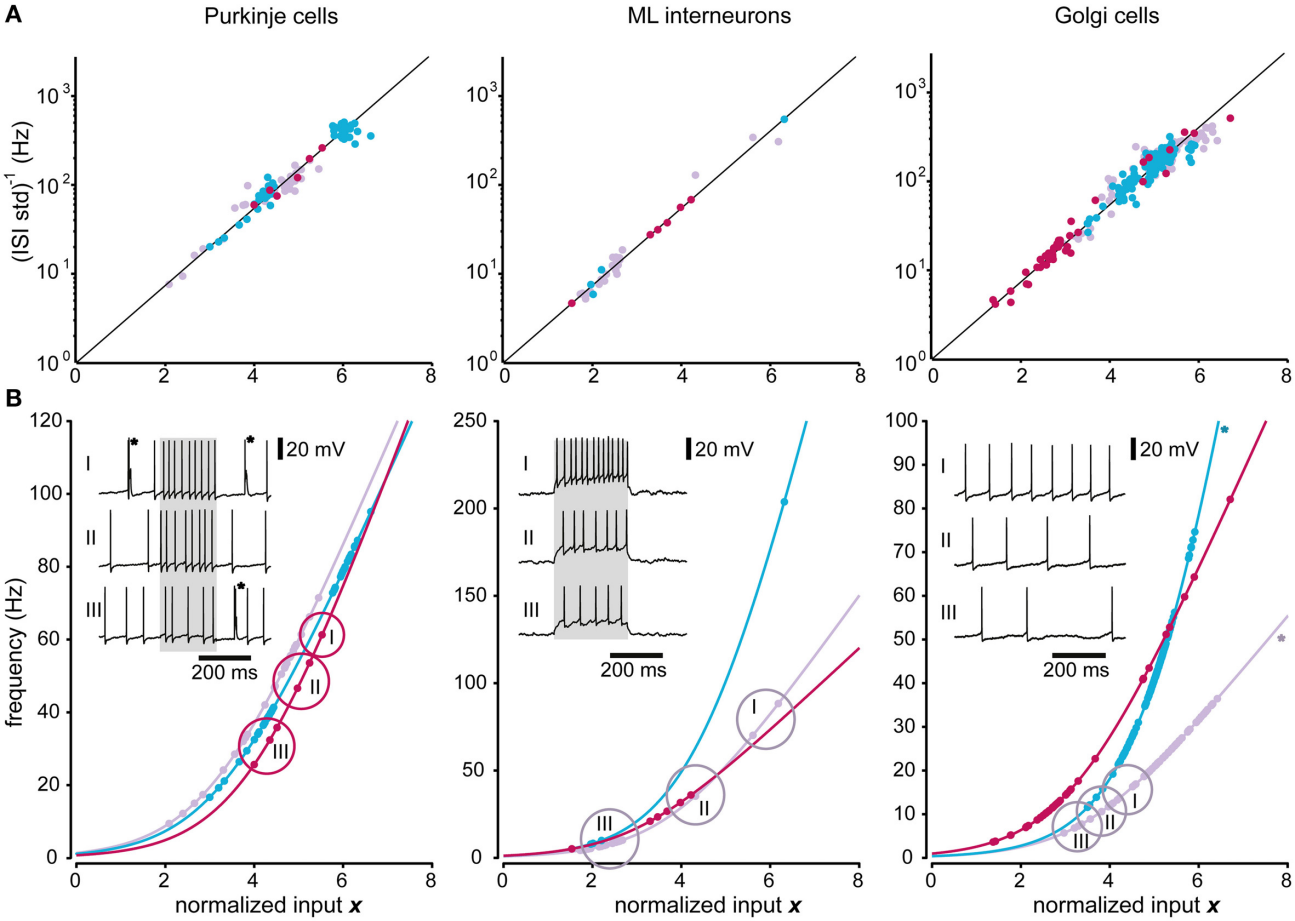
**Predictions of firing frequencies for neurons of the cerebellar cortex**. Similar display as in Figure 5 but for the three different neuron types recorded in the cerebellar cortex. **(A)** Relationship between the normalized input and the inverse of the standard deviation. **(B)** Relationship between the normalized input and the mean firing frequency. The two Golgi cells that had correlated residuals (DW-test, *p* < 0.05) are marked by asterisks. For the illustrated raw traces of Purkinje cells, the asterisks indicate complex spikes, which were not analyzed. The lower panels of each neuron type also contain three sample intracellular traces at different excitation levels as insets. The states that were found at each level are encircled and denoted with I, II, and III. The gray regions in the traces of the Purkinje cell and ML interneuron indicate the duration of the current pulses used.

Figure 6 illustrates the applicability of the model to the recorded Purkinje cells, molecular layer interneurons and Golgi cells. As for the spinal interneurons, the predicted relationship between input and the *i.s.d*. of the model provided a good fit also for the Purkinje cells (*p* > 0.01; DW-test; *R*^2^ = 0.94 ± 0.01; *N* = 3). The model accuracy, using AD-test was 94 ± 5% (*p* > 0.01; *N* = 85 stationary states). The model provided a good fit also for molecular layer interneurons (*p* > 0.01; DW-test; *R*^2^ = 0.99 ± 0.02; *N* = 3), with a model accuracy of 96 ± 7% (AD-test; *p* > 0.01; *N* = 34 states).

For the Golgi cells, the *F*-test of the residuals from the predicted relationship between input and *i.s.d*. failed for 2 out of the 3 neurons (*p* < 0.01; DW-test; *R*^2^ = 0.86/0.82), whereas it could not be rejected for the remaining neuron (*p* > 0.01; DW-test; *R*^2^ = 0.97). The model accuracy for the individual states was still quite high, 91 ± 5% (*p* > 0.01; AD-test; *N* = 241).

Finally, the residuals of all the linear regressions could not be rejected as normally distributed (*p* > 0.01, SW-test) and the 95% confidence intervals of the linear regression parameters all contained the predicted values (*p*_1_ = 1 and *p*_2_ = 0, see Materials and Methods).

**Figure 7:**
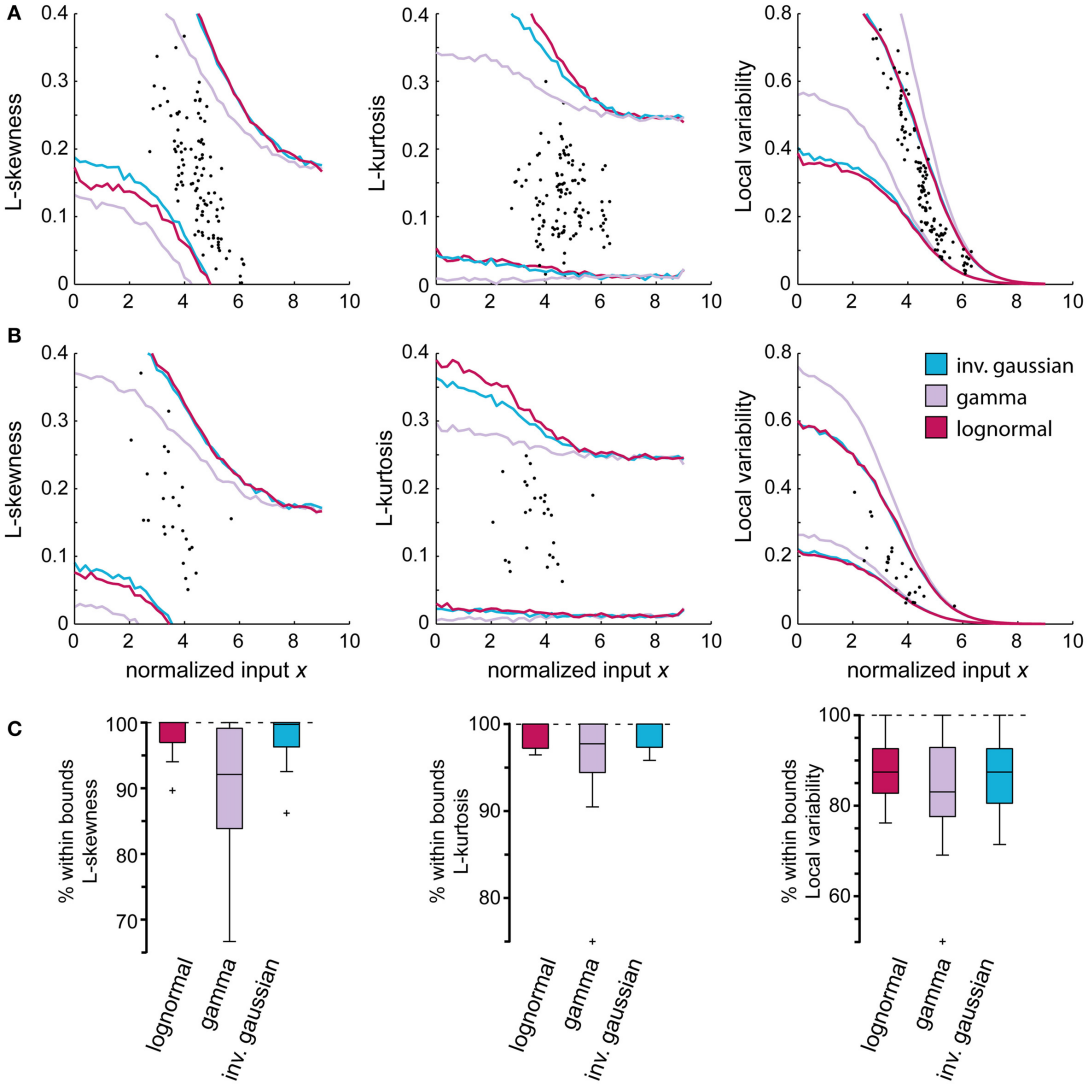
**Comparison between different probability density functions (PDFs) using higher order moments and the local variability (LV)**. Higher order moments of the empirical distributions and the local variability of the spike trains can be employed to validate the use of specific PDFs as approximations of the empirical distributions as they inform on features that cannot be seen using only the mean and standard deviation. **(A,B)** display the attained 99% confidence bounds of the constructed model using the gamma, lognormal and inverse Gaussian distribution. **(A)** Spinal interneuron that followed the predicted LV of the gamma distribution. **(B)** Representative spinal interneuron with no preferred PDF. **(C)** Summary of all tested neurons showing the proportion of stationary regions that fall within the confidence bounds of the respective model. The slight trend in **(B)** where the gamma distribution underestimates the L-skewness and L-kurtosis is more evident here, while there is no difference between the PDFs regarding the LV.

We also compared the accuracy and the higher order moments of the lognormal distribution with those obtained using the gamma and inverse Gaussian distributions. As shown in Figure 7, the approximations obtained using the latter two did not provide better predictions of the higher order moments of the empirical distributions. The accuracy (*p* > 0.01, AD-test) using the different distributions were also comparable. Using the lognormal distribution, 95 ± 5% accuracy was obtained, while the gamma distribution lead to an accuracy of 97 ± 3%, and the inverse Gaussian distribution to an accuracy of 95 ± 5%.

### Comparisons across neurons

**Figure 8:**
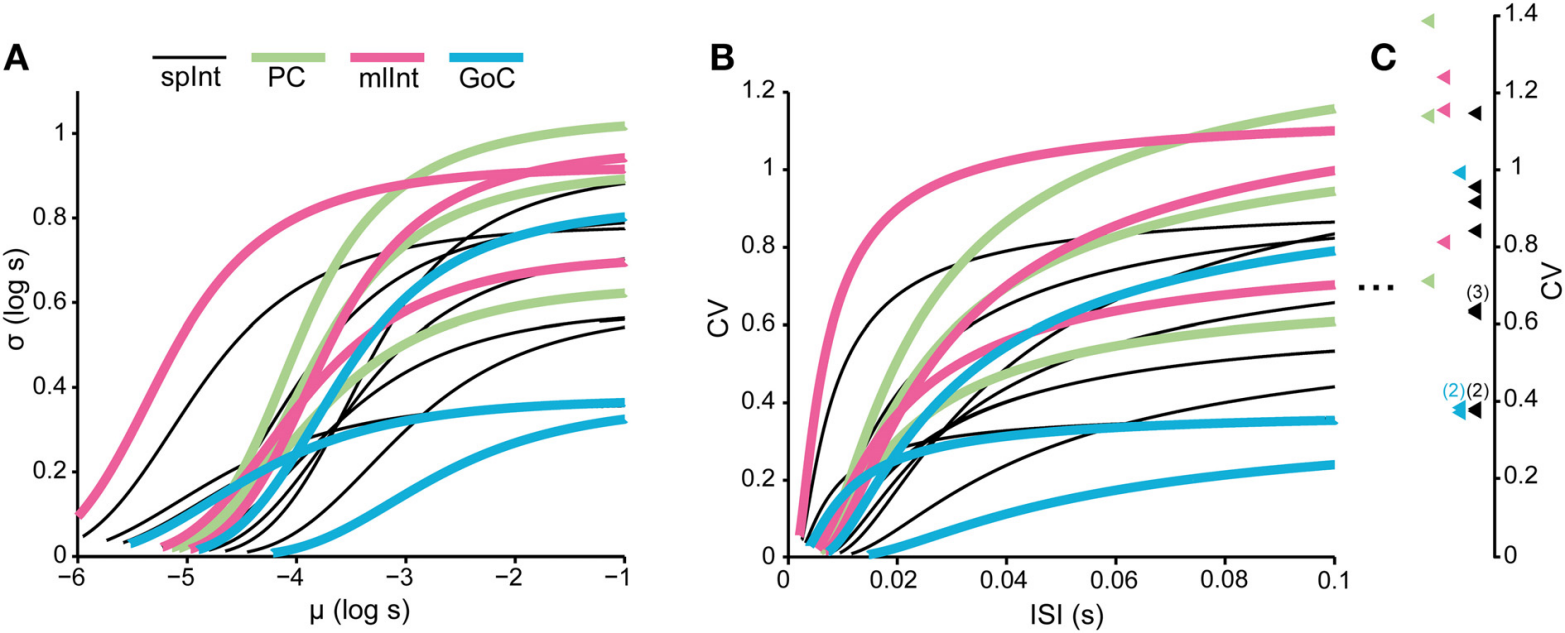
**Comparison between models of the different neuron types**. The figure illustrates that the curves of different neuron types overlap extensively, such that the difference between two neurons of the same type is similar to the difference of two neurons of differing types. **(A)** Parametric curves between the mean and the standard deviation of the log-normal distributions of the models. The range of the curves is limited by the range if the normalized input x, which is varied between 0 and 9. **(B)** The same relationship as in **(A)**, but now as the coefficient of variation (CV) in relation to the average ISI. **(C)** The colored triangles indicate the CV of the models as *x* → −∞ (see Table 1 for the formula). The numbers indicate whenever there is more than one neuron model whose asymptotic CV overlap in the figure.

It has previously been shown that it is possible to distinguish the type of neuron from its spontaneous firing using the firing rate and the entropy of the neurons spike train (Van Dijck et al., 2013). Note that the spontaneous firing in this case refers to the spontaneous activity at rest. In Figure 8, the models that were fitted to the neuron types in this study are compared to each other in order to investigate whether it is possible to differentiate the neurons types also outside of their resting state when they are driven to fire at other intensities. The analysis in Figure 8 is limited to the first two moments of the distributions, due to the short length of the spike trains, but the results indicate that there is no apparent relationship between the type of neuron and model behavior. Rather, the differences between neurons of the same type are comparable to the differences between neurons of different types. A consequence of this relationship, and the relationship between the firing frequency and the standard deviation of the ISIs described above, is that the spike firing statistics in the different neurons we explored here are apparently similar (or rather equally dissimilar) across neuron types when their input is modified so that they have similar firing rates.

### Model specificity

To rule out that the accuracy of the constructed models were not simply the result of over-fitting of random data or the possibility that any kind of unrelated ISI data would yield similar results, the accuracy of the models fitted to single neurons were compared to models that were fitted to 4, 50, and 100 stationary states randomly selected out of a total of 498 states (only states that could not be rejected as log-normally distributed, SW-test, *p* > 0.05) obtained from 45 different spinal interneurons with spontaneous activity (>2 Hz).

The proportion of the data points obtained from the randomly selected states that was accurately predicted by the model was on average 67 ± 13% (4 random states: 76 ± 28%, 50 random states: 67 ± 8%, 100 random states: 67 ± 5%; *p* < 0.01; AD-test), which was significantly lower than for the single neuron models (*p* < 0.01; student's *t*-test) for all neuron types. The distribution of *R*^2^ (4 states: *R*^2^ = 0.84 ± 0.25; 50 states: *R*^2^ = 0.88 ± 0.06; 100 states: *R*^2^ = 0.89 ± 0.02) was, just like the model fit, significantly different compared to the single neuron models (*p* < 0.01; student's *t*-test), except for the Golgi cell models.

### Input normalization

One requirement to perform the present analysis was to obtain long-lasting intracellular recordings under stable conditions *in vivo*, for which the whole cell recording technique was our method of choice. But for this approach there are sources of variability for the neuronal activity that could not be avoided, and which could change the relationship between the injected current and the average firing frequency. These sources are variations in background synaptic activity and variations in access resistance between the electrode and the cell. Although access resistance was compensated for off-line, this cannot be done with 100% accuracy and the error can vary over time. The variations in the background activity were outside of our control.

In order to validate the assumed linear relationship between the input current and the normalized input Equation (5c), the model was used to predict the normalized input from the experimental data. For each stationary state, both the *s.d*. and the frequency of the state were used to predict the normalized input through the inverse of Equations (6a,b). These predictions should be linearly related to the actual input current according to Equation (6c).

**Figure 9:**
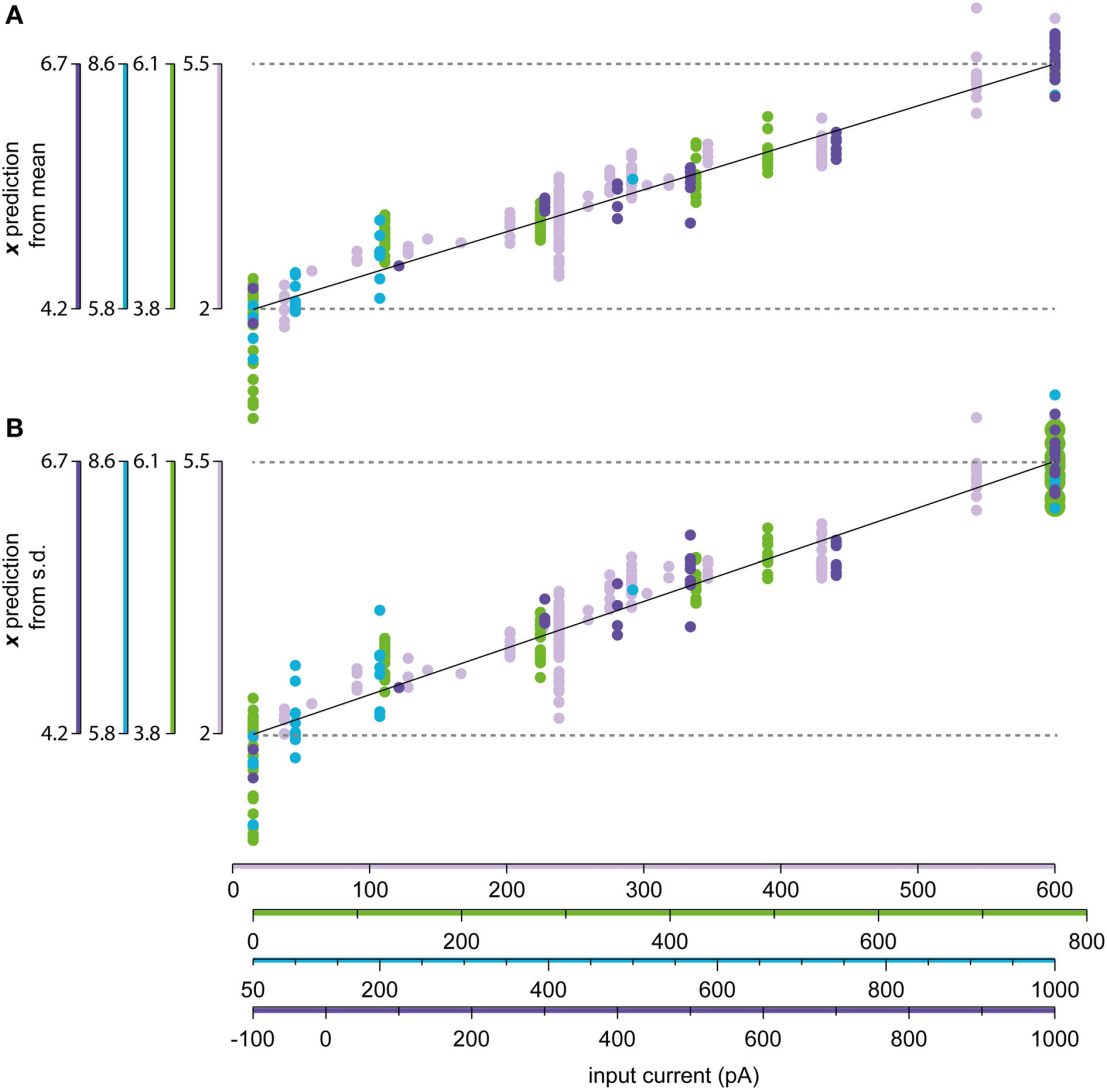
**Relationship between the injected current and the estimated input level**. **(A)** Linear regression of the relationship between the normalized input *x* and the injection current *I* in Equation (6c). The mean of the firing frequency was used to predict the value of *x* using the constructed model, which is compared to the recorded value of *I*. **(B)** Same as in **(A)**, but using the standard deviation of the ISIs instead of the mean as the predictor for *x*.

Figure 9 illustrates the relationship between injected current and the predicted normalized input for four sample neurons with a high number of stationary and log-normally distributed states with different current injections. The variability of the predicted input for each input current level is due to both the inherent stochasticity of the spike generation, but also the issues with background synaptic noise and variations in access resistance. However, overall there was a relatively good correspondence between the amount of injected current and the estimated normalized input, with the variability lying in a similar range as reported from other *in vivo* recordings (Nowak et al., 2003).

### Inhomogeneous model

A model of the firing statistics neurons across its potential range of stationary states can be extended to inhomogeneous situations with input that is not constant. The least complex model that does not incorporate transient behavior would use the log-normal hazard function in Equation (7) to describe the instantaneous firing rate.

(7) $$\lambda (t;\mu ,\sigma )=\frac{f(t;\mu ,\sigma )}{1-\Phi \left(\frac{\ln t-\mu }{\sigma }\right)}$$

where λ is the instantaneous firing rate, *f* is the log-normal PDF in Equation (1) and Φ is the cumulative density function of the standard normal distribution.

**Figure 10:**
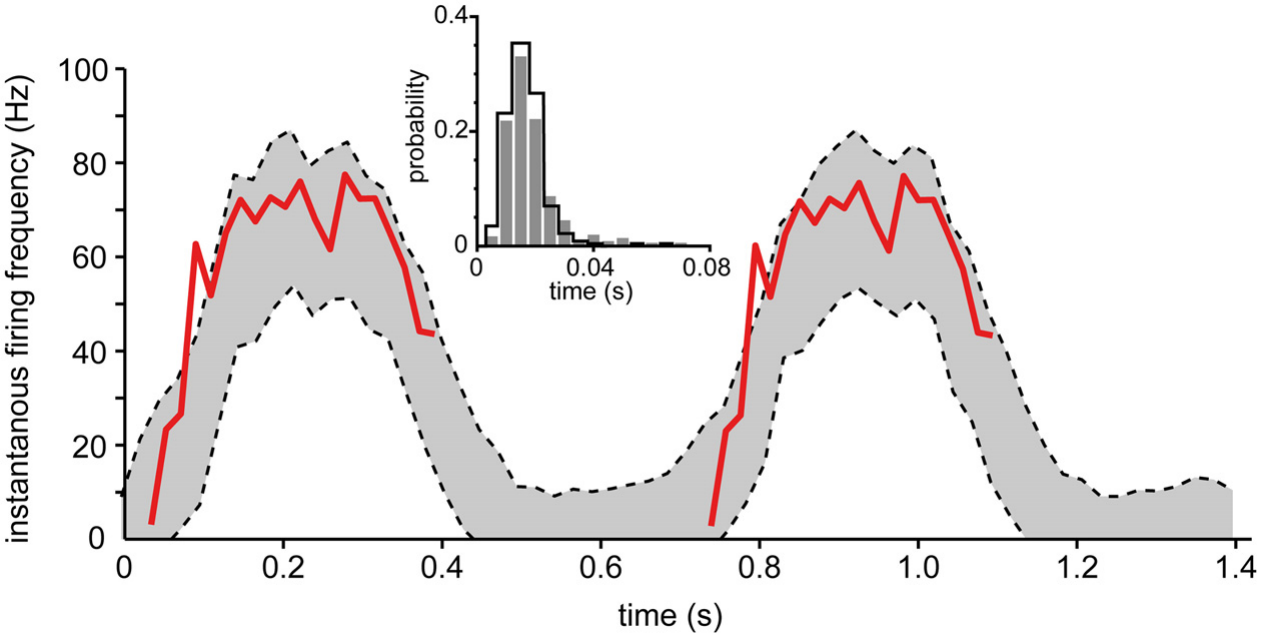
**Comparison of actual spike firing and spike firing predicted by the inhomogeneous model during fictive locomotion in spinal neurons**. The model took the intracellular membrane potential modulation of dorsal spinocerebellar tract (DSCT) neurons (Figure 3 of Fedirchuk et al., 2013) as the driving input and predicted the spike firing or the neuron using the inhomogenous model fitted to one of our spinal interneurons. The 95% confidence bounds predicted by the model (dashed lines) is shown and compared to the actual instantaneous firing frequency (red line) (Fedirchuk et al., 2013). Just like the experimental data, the two step cycles in the figure was binned into 30 bins each, where the data to each bin was computed from 12 simulations. This procedure was repeated 250 times to calculate the confidence bounds of each bin. (inset) Histogram showing the probability distribution of the ISIs from Fedirchuk et al. (2013) (solid curve), compared to those obtained through simulation (gray bars).

Intracellular recordings of the membrane potential as well as the spike firing during input modulation equivalent to that which would occur under behavior does to our knowledge not exist for any regular spinal interneuron *in vivo*, but does exist for the spinocerebellar tract cells of the lumbar cord. To demonstrate the applicability of our model in inhomogeneous situations, the parameters adapted to one of our spinal interneurons were used to simulate the firing during slowly modulated input that is seen during fictive locomotion in DSCT neurons (Fedirchuk et al., 2013). The recorded membrane potential, averaged across a number of cycles of locomotion, was used as the input to the modeled neuron (Fedirchuk et al., 2013). Figure 10 illustrates the fit between the output predicted by the model and the recorded instantaneous firing frequency. The actual firing almost completely stayed within the 95% confidence bounds, indicating that under these conditions the inhomogeneous model, generated from a spinal interneuron in the lower cervical segments, accurately predicts the actual firing behavior of DSCT neurons during fictive locomotion (with the exception of one data point of the actual spike firing that fell outside the confidence bounds of the model prediction, see Figure 10). The initial rise of the experimental response indicates a tendency of overshoot following the period of inactivity. Note however, that while the cyclic membrane potential modulations used as input and the instantaneous firing frequency were recorded from the same neuron (Fedirchuk et al., 2013), they were naturally not from the same step cycles. Due to this, some discrepancies between the predicted output by the model and the recorded frequency is expected.

## Discussion

We used long-term whole cell patch clamp recordings to characterize the neuronal spike firing statistics for a high number of stationary states across a wide range of membrane potentials *in vivo*. A striking finding was the strong relationship between the firing frequency and the stochasticity of the spike generation, which made it possible to predict the neurons' spike firing statistics across a continuum of input levels. Surprisingly, the differences between neurons of different types overlapped the differences between neurons of the same type. Based on our measurements, we formulated a model that captured the neurons' spike firing statistics with high accuracy across the operative range of membrane potentials, and included a simple inhomogeneous model for time-varying input. In a conductance-based modeling of neurons, this model can be used to comparatively easily solve the problem of how to introduce stochasticity in the spike generation (Goldwyn and Shea-Brown, 2011; Lim and Goldman, 2013). The advantage of this model is particularly the case where there is a dissociation between the membrane potential dynamics and spike initiation (Figures 1D,E; Naundorf et al., 2006). Even though Hodgkin-Huxley based models implementing synaptic noise have proven relatively successful in reproducing *in vivo*-like spike patterns (Gauck and Jaeger, 2000, 2003; Suter and Jaeger, 2004), this approach requires knowledge about the structure of the synaptic noise and will not capture spike generation that is dissociated from this process. Our model incorporates stochasticity both from synaptic noise, without requiring any knowledge about its structure, and from intrinsic, non-deterministic fluctuations in the spike generation.

The limitations of our findings lie primarily in possible errors in the estimate of the total actual input. However, the accuracy of the model predictions argued against that this was a problem of major importance. Also, the relationship between the firing frequency and the standard deviation lies beyond these measurement errors as they were based solely on the statistics of the stationary regions and not the actual input. In theory, one could have fitted the model to a neuron without artificial modulation (by current injection) of the total input if it spontaneously shifted between different stationary states. This is clearly one of the major advantages with the method we introduce: the statistics of the spike generation of individual neurons can be approximated from a limited amount of recorded spikes.

Why did the shapes of the curves describing the input-output relationship vary between neurons? A speculative answer is that there are differences in the actual resting membrane potential, the density of the voltage-sensitive ion channels between neurons and possibly variations in membrane capacitance. Importantly, these differences did not seem to depend upon the type of neuron. The parameters we tested for varied greatly within the same group of neurons (Figure 8), and the extent of the variability was overlapping between different types of neurons so that it was not possible to differentiate neuron types on basis of the model parameters. This was surprising, given that these types of neurons at least to some extent are known to vary with respect to the active conductances they express in their membranes (Mckay et al., 2005; Molineux et al., 2005; Forti et al., 2006; Zhong et al., 2010). One interpretation is that the spike generating mechanism relies on a limited set of ion channels (Schneidman et al., 1998; Fourcaud-Trocme et al., 2003; Naundorf et al., 2006; Saarinen et al., 2008; Stiefel et al., 2013), which is comparable across neuron types, but that the role of the bulk of the reactive membrane conductances is to shape the subthreshold responses of the membrane. In the case of neurons that have prominent contributions from such intrinsic, subthreshold membrane conductances, a modeling approach would need to implement the effect of these conductances separately, possibly as an autoregressive filter upon the input to the model, extending the linear input relationship that is currently used with temporal dynamics. Additional internal states could also be added to handle rebounds from e.g., Ca^2+^ concentration changes. The reason that our model still worked quite well suggest that the particular types of neurons we recorded from are not dominated by intrinsic conductances in the inactive *in vivo* state without anesthesia. This may also be a partial explanation for why the Golgi cells displayed the poorest model fit—Golgi cells are well known to have strong intrinsic conductances (Forti et al., 2006).

The parameters of our firing statistics characterization and model are not overtly related to any biophysical properties of the neuron, but were chosen to give closed form solutions to the f-I curve and its relationship to the standard deviation that provided a good enough fit. Other more detailed biophysical models exist, most prominently the leaky integrate-and-fire (LIF) model (Capocelli and Ricciardi, 1971). Compared to even more detailed models, the LIF model provides the advantage of analytical solutions that allows it to be used to approximate experimental ISI data (Rauch et al., 2003; La Camera et al., 2008). However, the actual effective values of the biophysical parameters that corresponded to the best ISI data fit of the LIF model were not correlated to measured values of membrane capacitance and time constant (Rauch et al., 2003; La Camera et al., 2006) and consequently best fits were found using parameters obtained through optimization to arbitrary values. In comparison, our model also relies on arbitrary parameters, but since these parameters have a phenomenological rather than biophysical origin (see Table 1 for examples) they are readily obtained from neuronal recordings in contrast to the LIF approach. For the relative simplicity and utility of the modeling of the spike generation, and to ensure a close link between the model and recorded neuronal spike firing statistics, our model hence offers substantial advantages.

Other generative phenomenological models of spike generation exist. In contrast to our model, they approximate spike trains with varying firing frequencies with inhomogeneous point processes where the parameters of the point process is varying with time and estimated from recorded spike trains. This method is performed using both Gamma distributions where both the shape and scale parameter varies with time (Shimokawa and Shinomoto, 2009) and Poisson processes (e.g., generalized linear models, Brunel et al., 2014). These models have in common the need to explicitly model the refractory period if the model should be able to capture such effects (Stevenson et al., 2008). This is true also when the escape rate is defined by the gamma distribution (Shimokawa and Shinomoto, 2009). By contrast, using the log-normal distribution, the refractory period is implicitly there due to its logarithmic definition, and by fitting it to a distribution of ISIs from a stationary state where the refractory period is present in the empirical distribution, it will naturally also be present in the fitted distribution. The current model is on the other hand limited from the lack of inhomogeneous input in the experimental setup.

Our model of spike generation was based solely on the spike firing statistics during stationary states. However, by the introduction of an inhomogeneous hazard function or escape rate, as described in the results, it was possible to also accurately predict the variation of spike firing across a range of membrane potentials that the neuron is likely to experience during ongoing behavior. In cases of extremely rapid changes in membrane potentials, such as can be induced by massive, synchronous activation of a large number of synapses that is often done artificially in *in vivo* recordings of neurons receiving sensory input, it is unlikely that our model would accurately capture the dynamics of the spike firing. On the other hand, at least for the relatively slow modulations of spike firing that spinal interneurons, Purkinje cells, Golgi cells and molecular layer interneurons display under behavior, such synchronous synaptic activation does not seem to occur and at least outside this range of activation dynamics our model is likely to generate accurate predictions. During asynchronous states (Renart et al., 2010), which may be the primary processing state of the neocortex, neocortical pyramidal neurons may obey similar rules of spike generation since also these neurons appear to work primarily as rate coders (Priebe et al., 2004; London et al., 2010). The challenge for a corresponding analysis of neocortical neurons lie in finding stationary states in the face of the up-down states these neurons display *in vivo*. This work shows that it is possible to obtain good approximations of any neuron's spike generation under rate modulated firing using phenomenological observations of the spike firing statistics that are obtained by recording the neurons from a few different stationary states.

### Conflict of interest statement

The authors declare that the research was conducted in the absence of any commercial or financial relationships that could be construed as a potential conflict of interest.
